# A Solitary, Large Calcaneal Osteochondroma Growing Extensively After Skeletal Maturity: A Case Report and Review of the Literature

**DOI:** 10.7759/cureus.42570

**Published:** 2023-07-27

**Authors:** Kyriakos Avramidis, Constantinos Katounis, Panagiotis Krikis, Pavlos Skoufogiannis

**Affiliations:** 1 Department of Orthopaedics, General Hospital of Volos, Volos, GRC; 2 Department of Radiology, General Hospital of Volos, Volos, GRC; 3 Department of Pathology, General Hospital of Volos, Volos, GRC

**Keywords:** foot and ankle tumors, calcaneal chondrosarcoma, osteochondroma imaging, calcaneal osteochondroma, osteochondroma

## Abstract

Although osteochondromas are the most common benign tumors in the skeleton, calcaneal osteochondromas are very rare. New onset of pain or rapid growth of the tumor, especially after the closure of the epiphyseal growth plate, might reflect malignant transformation. However, enlargement of solitary osteochondromas reported as benign in a skeletally mature patient is present in the literature. We report the clinical and radiologic findings of a calcaneal osteochondroma with an extremely rare placement and painful rapid growth causing limited ambulation in a 27-year-old male. After surgical removal of the tumor, histologic examination demonstrated no evidence of malignancy, and there was no recurrence during the three-year follow-up.

## Introduction

Osteochondromas are the most common benign bone tumors. They are sessile or pedunculated projections on the external bone surface, typically identified in patients younger than 20 years of age [1]. Most osteochondromas (85%) present as solitary nonhereditary lesions; 15% occur in the context of hereditary multiple exostoses (HME), a genetic disorder inherited in an autosomal dominant manner and associated with germline mutations in the tumor-suppressor genes EXT1 or EXT2 in almost 90% of cases [1]. Osteochondromas may occur on any bone in which endochondral ossification develops [1]. They usually occur in the metaphyses of the long bones and are most commonly observed around the knee [2]. Foot and ankle osteochondromas are uncommon. They are typically identified earlier than in other regions since their more superficial placement in the foot renders a growing osseous mass more obvious and symptomatic [3].

The tumor increases in size throughout childhood, and its growth usually stops after the closure of the epiphyseal plates [1]. Growth or radiologic alteration of an osteochondroma in adulthood may suggest malignant transformation into chondrosarcoma [1,4]. Malignant transformation is documented to occur in almost 1% of solitary osteochondromas and 10% of HME cases [1,3,5]. The lesion is usually a low-grade chondrosarcoma [4] and less often a secondary osteosarcoma [2]. However, extensive growth of an osteochondroma into adulthood without malignant transformation has been reported in the literature [3, 5-8].

We report the case of a solitary calcaneal osteochondroma in a skeletally mature patient. The lesion demonstrated extensive growth long after the closure of the epiphyseal growth plates, causing difficulty walking, and was eventually removed. Histologic examination did not show malignancy, and there was no recurrence of the tumor three years following its excision.

## Case presentation

A 27-year-old, otherwise healthy male, presented with a seven-year history of a bulging mass in the plantar-lateral hindfoot area. This firm nodular lesion demonstrated most of its growth over the previous three years and was associated with increasing discomfort in walking and prolonged standing. The overlying skin appeared stretched without ischemic pigmentation and demonstrated an area of hyperkeratosis in the posterior plantar area. The heel was tender on deep palpation. The ankle and hindfoot joints demonstrated a full range of motion without any stiffness.

**Figure 1:**
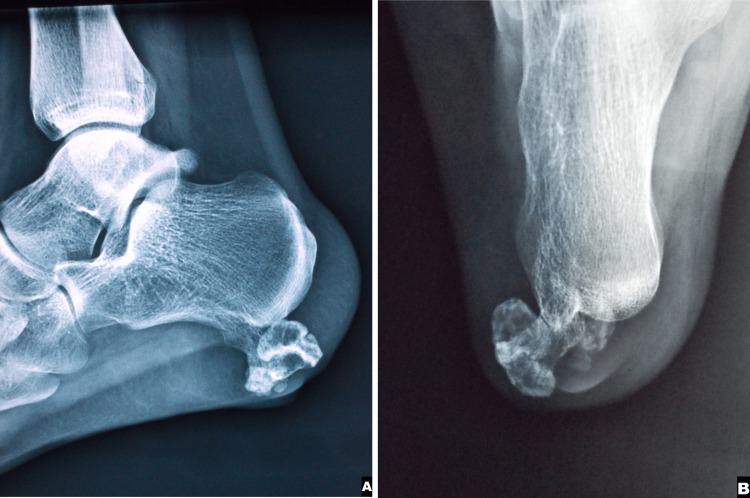
Lateral and axial ankle views Figure 1A: A lateral X-ray demonstrating the posteroinferior location of the tumor and its large exophytic osseous growth; Figure 1B: An axial radiograph reveals its origin in the lateral process of the calcaneal tuberosity. Appearance is compatible with an osteochondroma.

Plain radiographs showed a large exophytic pedunculated bone mass originating from the lateral process of the calcaneal tuberosity (Figures 1A, 1B).

**Figure 2:**
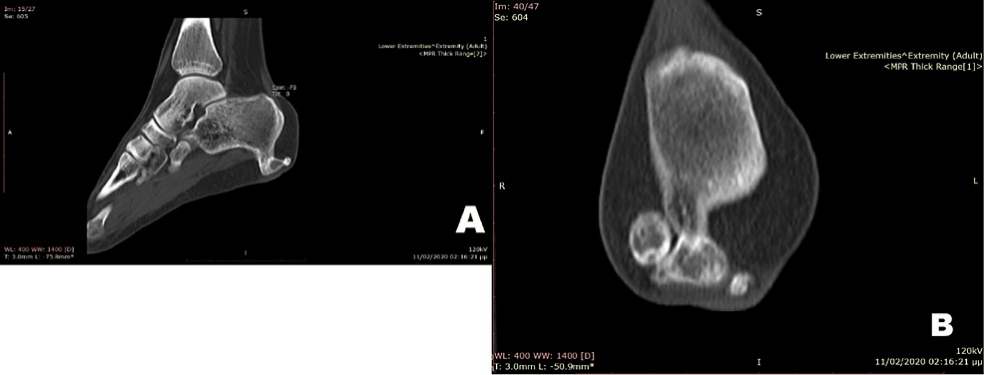
A preoperative CT scan of the affected foot A preoperative sagittal (Figure 2A) and coronal (Figure 2B) CT scan of the foot without contrast material Images demonstrate a pedunculated osteochondroma with no visible cartilage cap. Measured dimensions: anteroposterior 45 mm, superoinferior 25 mm, and mediolateral 30 mm

The tumor, measuring 4.5 cm in its greatest dimension, extended into the calcaneometatarsal ligament at the lateral aspect of the heel. A thorough radiological skeletal survey was conducted with X-rays of all long bones, pelvis, and spine, revealing the absence of other osteochondromas and excluding in this way the diagnosis of HME. Computed tomography (CT) demonstrated appearances typical of an osteochondroma and revealed a complete closure of the growth plates (Figures 2A, 2B).

The tumor demonstrated a distinct margin with no visible cartilage cap, features indicative of the benign nature of the lesion. However, a secondary chondrosarcoma diagnosis could not be ruled out, given its significant growth into adulthood and the large size of the tumor.

**Figure 3:**
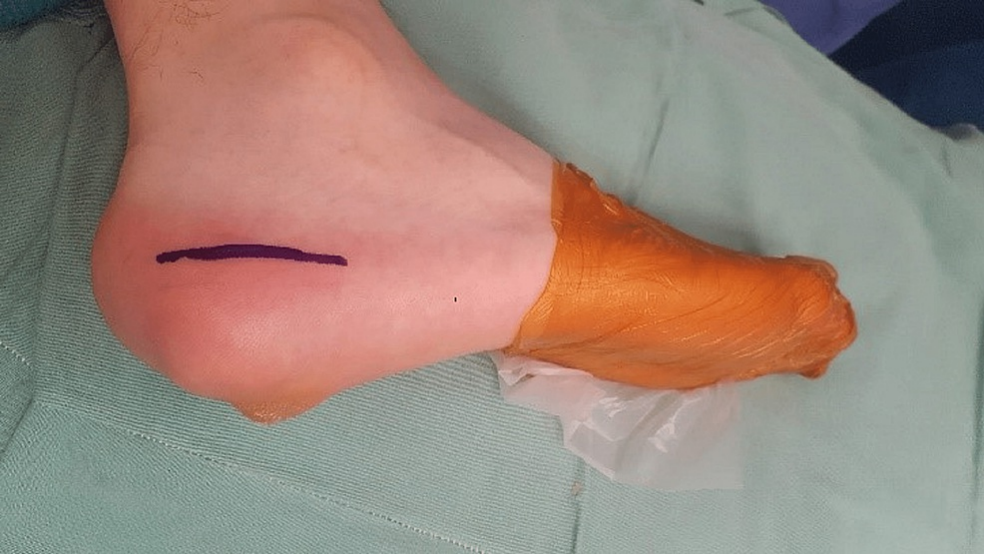
Patient positioning and the marking of the incision Patient is in the lateral decubitus position. The mark indicates the incision length (approximately 4 cm) at the border of the thick plantar skin with the normal lateral skin.

**Figure 4:**
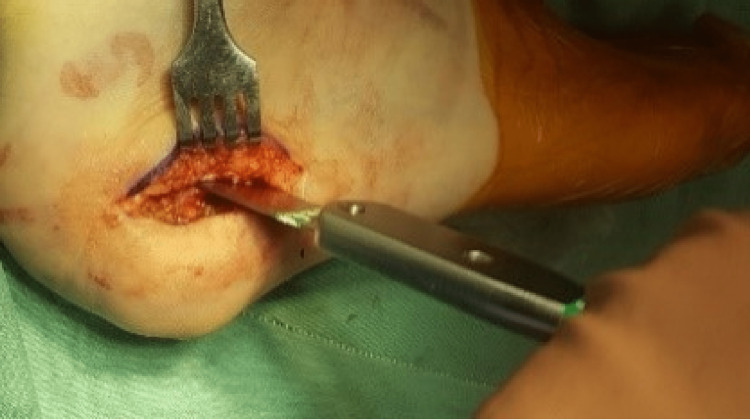
Resection of the tumor Resection of the tumor on healthy margins by the use of a thin osteotome.

With the patient placed in the lateral decubitus position (Figure 3), extensive resection and osteotomy of the lateral process of the calcaneal tuberosity were performed on healthy margins (Figure 4).

Particular care was taken to preserve the insertion of the long plantar ligament and most of the calcaneometatarsal ligament insertion on the calcaneal tuberosity, as well as to avoid injury to the abductor digiti minimi and/or its nerve.

**Figure 5:**
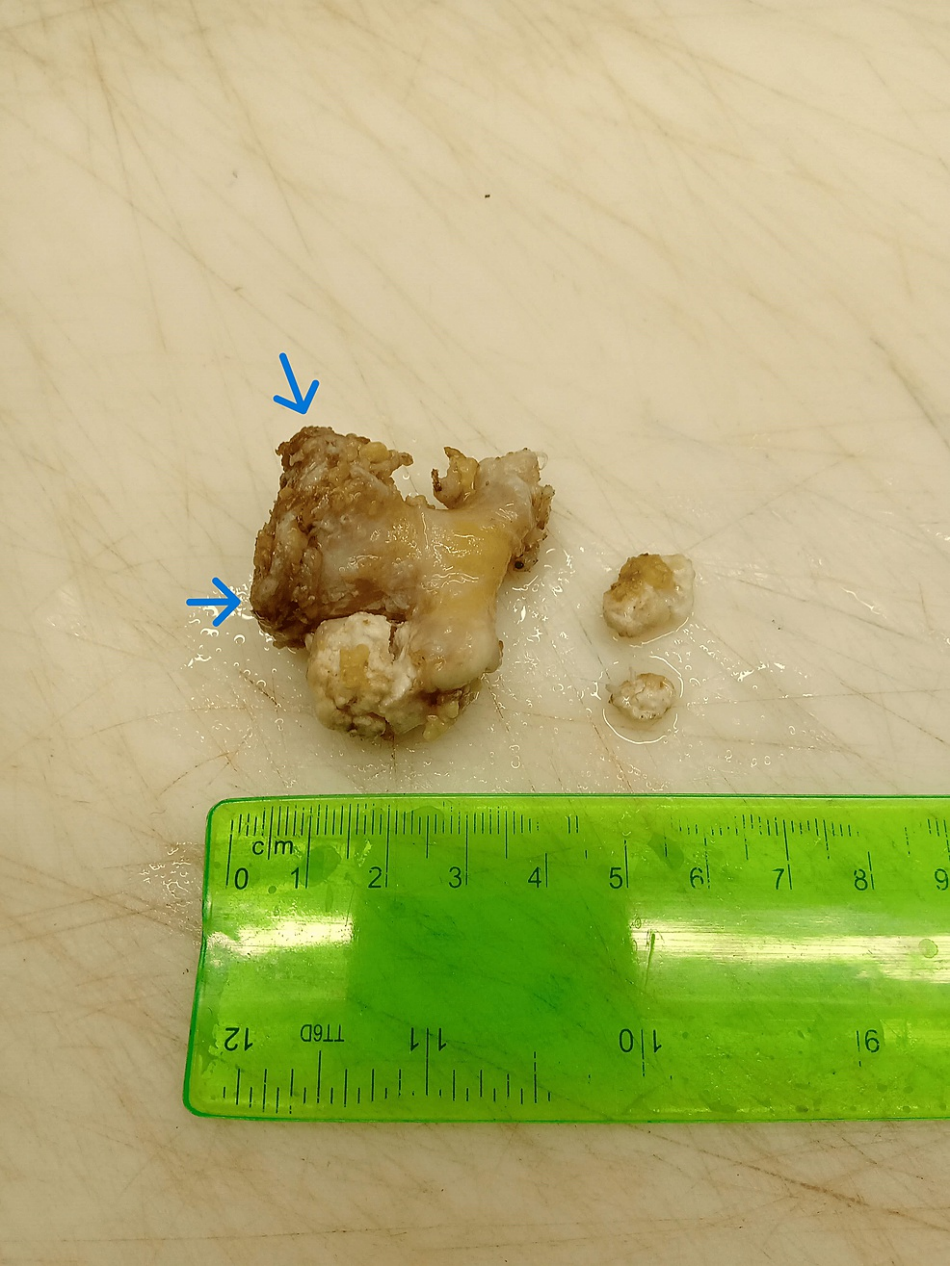
Resected tumor Measurement and preparation of the tumor for histopathologic examination. The maximum cartilage cap (arrows) thickness measured is 8mm.

After resection of the tumor, measurements revealed a rather sizeable lesion of 45mm x 30mm x 25mm in maximum dimensions (Figure 5).

**Figure 6:**
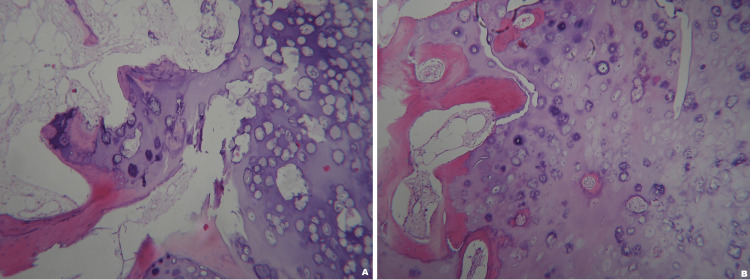
The microscopy of the resected tumor Figures 6A-6B: The lesion is composed of a proliferating cartilaginous cap covered by fibrous perichondrium overlying a bony stalk. The chondrocytes in the cartilage cap have a very characteristic columnar arrangement toward the base, where enchondral ossification occurs. The appearance is similar to that of an epiphyseal plate. Η&Ε x 100 H&E: hematoxylin and eosin stain

**Figure 7:**
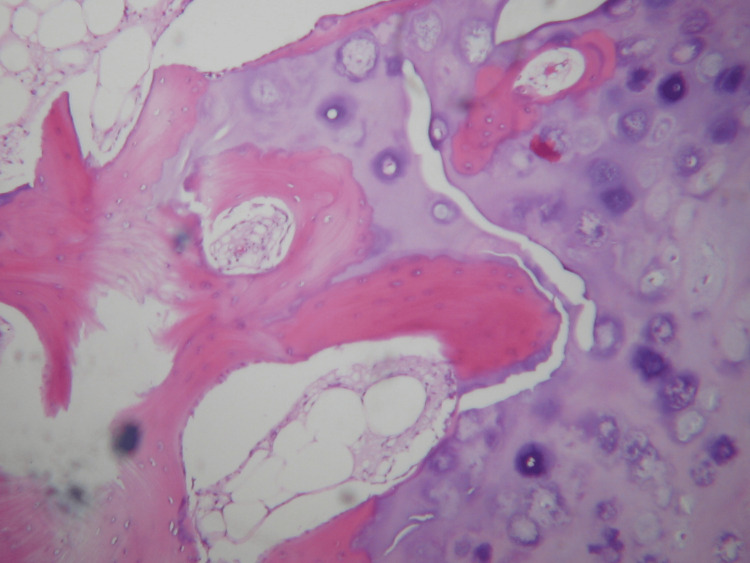
High-magnification microscopy of the specimen High-magnification of the junction where the cartilaginous cap merges into the underlying spongiosa. It is not unusual to see residual islands of cartilage in the middle of bony trabeculae in the stalk. This appearance is not indicative of invasion and does not suggest malignancy. Η&Ε x 200 H&E: hematoxylin and eosin stain

The histologic examination showed architecture typical of an osteochondroma without the morphologic features of a secondary malignant transformation. The cartilage appeared to merge with bony trabeculae with intervening marrow space. Chondrocytes were arranged in clusters, demonstrating no significant atypia or definitive myxoid change (Figures 6A, 6B, 7).

**Figure 8:**
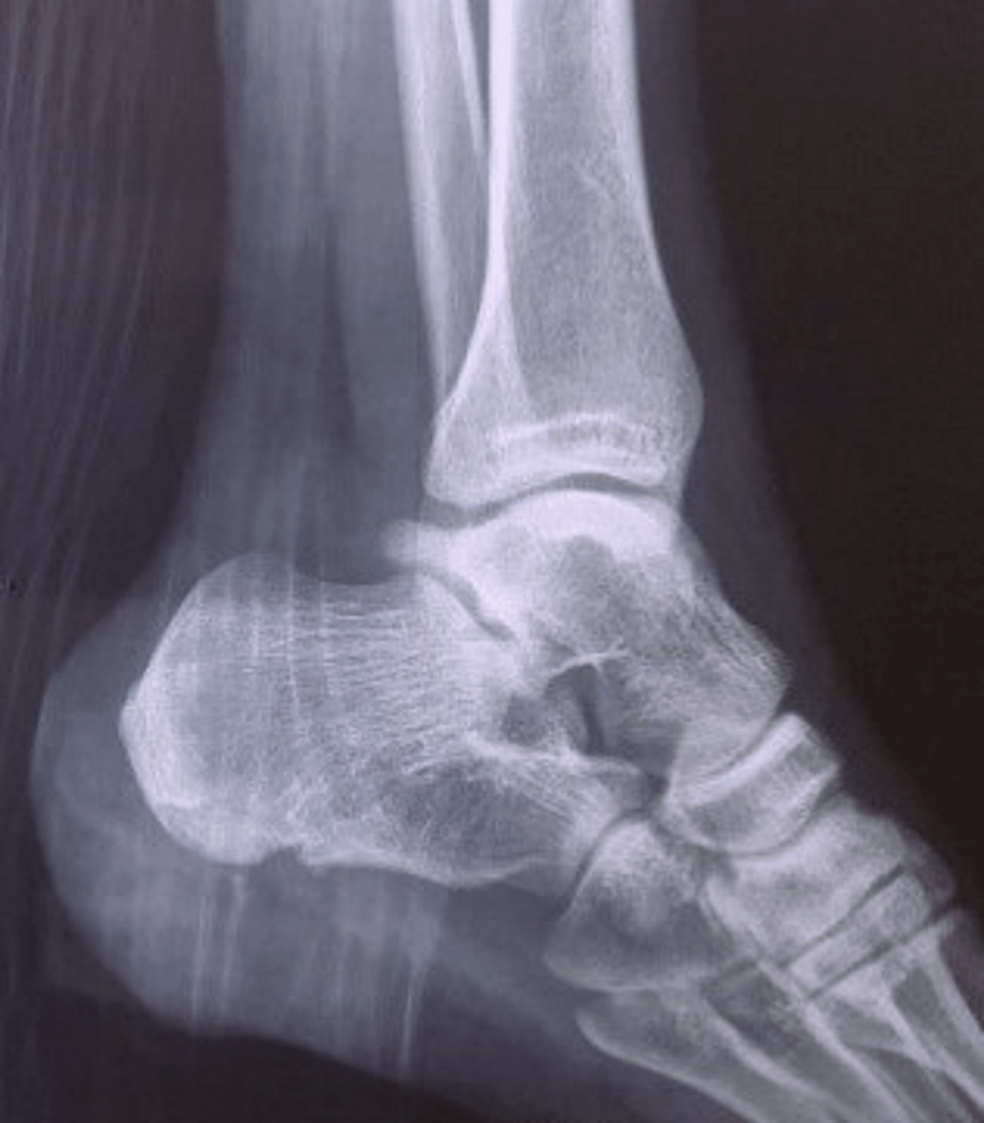
The patient's three-year postoperative follow-up X-ray

The postoperative course was uneventful. The surgical wound healed without complications, and the patient's gait was restored to normal after a four-week abstinence from weight bearing. On the patient’s last follow-up visit, three years post-excision, no clinical or radiological recurrence of the tumor was revealed (Figure 8).

## Discussion

Only 10% of osteochondromas occur in the small bones of the hands and feet, including the calcaneus [5,7]. Subungual exostoses, considered to be osteochondroma variants, are the most common occurrences in the aforementioned anatomic areas [1,9]. These osteocartilaginous tumors affecting the distal phalanx of the toes and fingers (most frequently the first toe) share some radiographic features with osteochondromas but pathologically represent a distinct entity [10].

The growth of osteochondromas usually parallels that of the patient, and the lesion often becomes quiescent when the epiphyses have closed. At skeletal maturity, any symptoms observed are typically related to mechanical stress such as nerve and/or muscle compression, bursa formation, popliteal pseudoaneurysm or vein thrombosis, fracture through the peduncle, and infarction within the tumor mass [5]. New onset of pain in a formerly stable lesion, accelerated growth, a large size of the lesion, and growth beyond skeletal maturity may indicate malignant transformation [3,11]. 

Chondrosarcoma is the most frequent malignant tumor arising from osteochondroma [4,12]. Secondary chondrosarcomas occur at a younger age than primary ones, more frequently in males, and have a predilection for flat bones [12]. Willms [13] reported a case of malignant transformation of a pelvic osteochondroma in a patient with multiple cartilaginous exostoses. Nonetheless, any bone with a true osteochondroma may be at risk. The report by Malik et al. [11] describes the case of a solitary calcaneal osteochondroma transforming into a chondrosarcoma. Garrison et al. [4], in their review of 75 cases of secondary chondrosarcoma stemming from an osteochondroma, identified only one chondrosarcoma of the calcaneus in a patient with HME.

Simon and Springfield [14] suggest that the incidence of malignant transformation into chondrosarcoma is directly analogous to the cartilage volume in the underlying preexisting osteochondroma. Cartilage cap thickness greater than 2 cm in adults and 2 cm to 3 cm in growing children is indicative of malignant degeneration [9, 14, 15].

Diagnostic investigation of osteochondromas includes plain radiographs (often diagnostic on their own) and additional imaging modalities such as CT, MRI, or bone scanning to determine surgical planning and/or exclude sarcomatous degeneration [3].

A CT scan is helpful in planning surgery by showing anatomic relationships with reasonable accuracy, but it is inaccurate in detecting and measuring cartilage thickness [16], an important criterion for malignant transformation [14]. In our case, the CT scan failed to demonstrate the presence of a cartilage cap; however, after the excision of the lesion, the true cartilage thickness of the specimen ranged from 5 cm to 8 mm.

An MRI demonstrates the extent of the lesion, possible soft tissue involvement, and cartilage cap thickness and location. A thick cartilage cap demonstrating high signal intensity on T2 images may suggest a malignant transformation [17].

On bone scintigraphy, osteochondromas may appear as focal uptake of radionuclides adjacent to the growth plate, especially in patients whose skeleton has not yet reached maturity. Stable lesions in adults may not show any radionuclide uptake. Planar bone scintigraphy is useful for detecting asymptomatic deep-seated lesions, especially in the context of HME; however, this investigation is not specific for distinguishing between osteochondromas and malignant chondrosarcomas [18].

Symptomatic tumors constitute an indication for surgical excision, and, in the case of larger lesions raising suspicion of malignant transformation, a biopsy and histopathological examination should precede radical resection [3].

Excision with a tumor-free resection margin is the treatment of choice. Complete tumor resection is associated with a local recurrence rate of less than 2%. In the case of incomplete excision, any cartilage cap remnants may result in recurrence [3]. In our patient, one-stage removal was decided based on the radiological characteristics of the benign nature of the lesion and the absence of HME. On histological examination of the specimen, the thickness of the cartilage cap varied from 5 mm to 8 mm, its normal architecture was preserved, and there was no evidence of tumor infiltration into soft tissues.

Krieg et al. [6] were the first to describe the extensive growth of a solitary osteochondroma of the proximal end of the fibula in a skeletally mature male patient without malignant transformation. A small tumor of the proximal fibula was incidentally identified in a patient at the age of 17, grew much larger, and became symptomatic at the age of 25, causing persistent pain and paresthesia in the distribution of the tibial nerve. Suspicion of malignancy was raised; however, surgical excision of the lesion followed by histological examination demonstrated a benign osteochondroma. The patient's follow-up lasted 42 months, and no evidence of recurrence or metastasis was noted.

Overall, calcaneal tumors and tumorous entities are relatively rare, comprising approximately 3% of foot and ankle osseous lesions [1]. Differential diagnosis includes benign and malignant primary bone tumors as well as a spectrum of reactive bone disorders, such as Turret’s exostosis, bizarre parosteal osteochondromatous proliferation (BPOP), and florid reactive periostitis [3, 9]. Calcaneal spurs are traction lesions located at the site of insertion of the plantar aponeurosis and are not true osteochondromas [3].

Only a few reports of calcaneal osteochondroma exist in the literature [3,5,7,8,11,19,20]. In these articles, the exact tumor location varies. It has been described at the inferior medial tubercle [3], posterior to the ankle joint [8], posteroinferior to the medial malleolus [7], involving the plantar body [19], and in our case, similarly to Nogier et al. [5], originating from the plantar and lateral aspects of the calcaneus. Karakurum et al. [20] reported a case of bilateral symmetric osteochondromas of the peroneal tubercle in a 24-year-old woman, discovered when the left side lesion became symptomatic, and thus it was excised. It is unclear whether this last case is one of a true osteochondroma or simply of hypertrophied tubercles.

Symptomatic growth of calcaneal osteochondroma in a skeletally mature patient without malignant change was demonstrated in some of the above articles [3, 5, 7, 8]. Blitz et al. [3] reported a large osteochondroma of the inferior medial tubercle of the calcaneus in a 40-year-old female with a six-month history of painful growth. A biopsy of the tumor confirmed its benign nature, and 3.5 years after its complete excision, no recurrence was noted. However, this patient never regained her previous level of activity.

Nogier et al. [5] reported on another large calcaneal osteochondroma involving the inferolateral aspect of the calcaneus in a 36-year-old man. Similar to our patient, this case also involved extensive growth in adulthood without malignant transformation or recurrence four years post-excision.

Koplay et al. [7] described the case of an osteochondroma arising from the posteromedial calcaneus in a 25-year-old female demonstrating late rapid growth. Recurrence occurred following surgical excision, and the tumor was re-excised six months later. Pathologic examination once again revealed a benign osteochondroma. No recurrence of the tumor was detected in the nine months following its second excision.

Kumar et al. [8] presented a case of symptomatic retrocalcaneal bursitis in a 58-year-old farmer due to the late growth of a posteromedial calcaneal osteochondroma. The tumor and the inflamed bursa were removed. There was no evidence of malignancy on histopathological examination, and no recurrence occurred six months following the operation.

Our report is another case of extensive growth of a calcaneal tumor after epiphyseal plate closure, which, following excision, proved to be a benign osteochondroma on histopathological examination. The patient returned to his previous level of activity soon after the operation, and no recurrence of the lesion was demonstrated after monitoring him over a three-year period. Ours is one of the longest-lasting follow-up periods cited in the relevant literature, along with the reports of Blitz et al. [3] and Nogier et al. [5].

## Conclusions

Benign osteochondromas can grow in size and become symptomatic in skeletally mature patients without malignant transformation. Calcaneal osteochondromas are rare conditions. Large lesions are more likely to cause symptoms, necessitating a radiological investigation and resection of the healthy bone margin. Remnants of the cartilage cap after incomplete excision may result in tumor recurrence, especially in growing lesions.
